# Network pharmacology and experimental evidence reveal the protective mechanism of Yi-Qi Cong-Ming decoction on age-related hearing loss

**DOI:** 10.1080/13880209.2022.2101671

**Published:** 2022-08-07

**Authors:** Yi-Fang Yang, Xi-Rui Yan, Rui-Xin Wu, Ning Li, Min Chu, Yang Dong, Shu-Ping Fu, Jian-Rong Shi, Qing Liu

**Affiliations:** a School of Basic Medical Sciences, Shanghai University of Traditional Chinese Medicine, Shanghai, China; b Experimental Teaching Center, Shanghai University of Traditional Chinese Medicine, Shanghai, China; c Key Laboratory of Acupuncture and Medicine Research of Ministry of Education, Nanjing University of Chinese Medicine, Nanjing, China

**Keywords:** Molecular docking, molecular dynamics simulation, HEI-OC1, apoptosis, mitochondrial membrane potential, AKT1

## Abstract

**Context:**

Yi-Qi Cong-Ming (YQCM) decoction has been widely used to prevent age-related hearing loss (ARHL), the most prevalent neurodegenerative disease in the elderly.

**Objective:**

To explore the mechanism of YQCM decoction in the treatment of ARHL.

**Materials and methods:**

The chemical constituents of YQCM were screened from the Traditional Chinese Medicine Systems Pharmacology Database. Potential targets of YQCM against ARHL were predicted by DrugBank, GeneCards, and OMIM database. Protein-protein network and enrichment analysis were used for exploring possible molecular mechanisms. Molecular docking and an *in vitro* model of ARHL by exposing auditory cells with 100 μM H_2_O_2_ for 3 h were applied. Cell viability and mitochondrial membrane potential (ΔΨM) were detected by CCK-8 and high-content analysis. γH2AX and cleaved caspase-3 were detected by Western blot.

**Results:**

The main compounds have good affinities with hub targets, especially AKT1, PTGS2, and CASP3. GO and KEGG analysis showed that the main biological process and key targets were related to negative regulation of the apoptotic process. H_2_O_2_ treatment could reduce the cell viability by 68% and impaired ΔΨM, while 90 μg/mL YQCM pre-treatment could restore the cell viability by 97.45% and increase ΔΨM (2-fold higher). YQCM pre-treatment also reduced γH2AX and cleaved caspase-3 protein levels.

**Conclusions:**

Our study suggested that YQCM prevents ARHL by modulating the apoptosis process in auditory hair cells. Moreover, this study proved that bioinformatics analysis combined with molecular docking and cell model is a promising method to explore other possible pharmacological interventions of ARHL.

## Introduction

Age-related hearing loss (ARHL), also called presbycusis, is one of the universal features of mammalian ageing which is characterised by gradual, progressive, bilateral loss of hearing starting from a high-frequency region of the hearing spectrum (Yamasoba et al. 2013). It is the third leading cause of chronic disability in older adults (Livingston et al. 2017; Loughrey et al. 2018) and influences approximately one-third of people aged over 65 (Patel and McKinnon 2018). Hearing loss not only affects the hearing quality, but is also associated with loneliness, social isolation (Maharani et al. 2019), and an increased risk of cognitive decline in older adults (Lin and Albert 2014; Jafari et al. 2019; Livingston et al. 2020). The most common method of hearing rehabilitation in presbycusis is the prescription of hearing aids and cochlear implantations. Hearing aids are designed to replace the amplification and compression that are no longer provided by the outer hair cells, the sensory-motor cells of the mammalian cochlea. This approach can improve the perception of weak sounds in quiet environments. However, for high-intensity sounds in background noise, the aids often fail to restore the perception of speech (Armstrong et al. 2022). Therefore, only a small fraction (10–20%) of older people with significant impairment use a hearing aid (McCormack and Fortnum 2013). Although cochlear implants can bypass damaged hair cells by providing direct electrical stimulation of spiral ganglion neurons (SGNs), the beneficial effects of cochlear implants are also strongly limited in elderly populations due to SGNs degeneration (Shibata et al. 2011). Apart from these challenges, the high cost of cochlear implants also imposes a significant financial burden on users. Thus, it is urgent to make efforts in pharmacological prevention and treatment to delay the occurrence and development of ARHL

**Figure 1.**
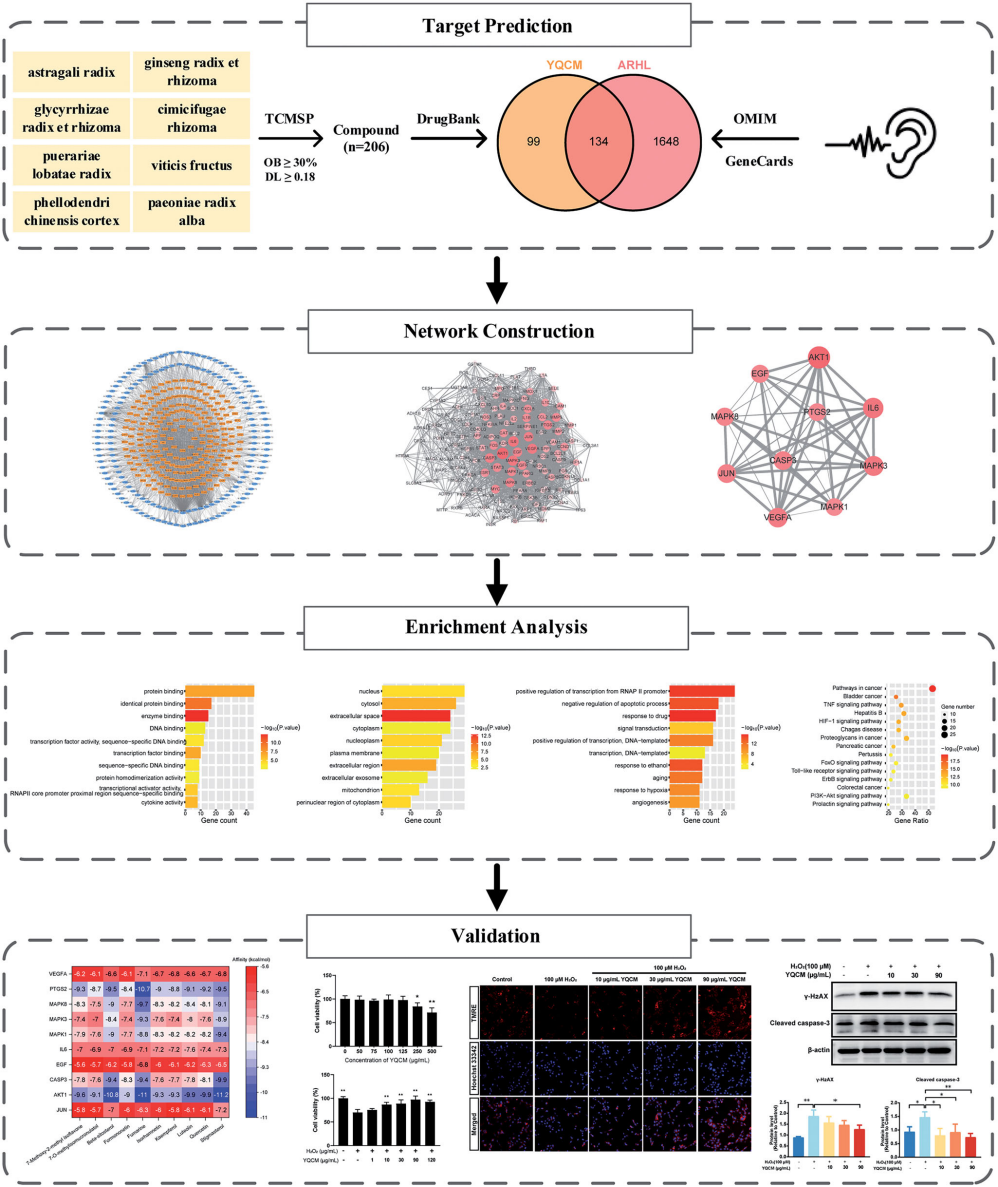
The flowchart of the technical strategy used in this study. Experimental methods include target prediction for YQCM’s compound and ARHL, construction and topological analysis on compound-target network and PPI network, enrichment analysis on GO and KEGG pathway, molecular docking, and *in vitro* study.

During the process of ARHL, three dominant morphological changes occur in the ageing cochlea: (1) the loss of sensory hair cells, which are responsible for the conversion of sound stimulations to neural impulses; (2) the loss of SGNs, which serve as the primary carrier of auditory information; (3) the atrophy of the stria vascularis (SV) of the cochlear lateral wall of scala media, which maintains endolymph potential (Keithley 2020). The exact mechanisms behind morphological changes are still being explored, but over time more evidence has come to light supporting the theory that oxidative stress (Tavanai and Mohammadkhani 2017; Pak et al. 2020) and inflammation (Verschuur et al. 2012; Uraguchi et al. 2021) incur the deleterious effects leading to this damage. Yi-qi Cong-ming (YQCM), a famous decoction composed of 8 herbs including Ginseng radix et rhizome (*Panax ginseng* C.A.Mey. [Araliaceae]), Astragali radix (*Astragalus mongholicus* Bunge, [Fabaceae]), Glycyrrhizae radix et rhizome (*Glycyrrhiza uralensis* Fisch. ex DC. [Fabaceae]), Cimicifugae rhizome (*Actaea cimicifuga* L. [Ranunculaceae]), Puerariae lobatae radix (*Pueraria montana var. lobata* (Willd.) Maesen & S.M.Almeida ex Sanjappa & Predeep [Fabaceae]), Viticis fructus (*Vitex trifolia* L. [Lamiaceae]), Paeoniae radix alba (*Paeonia lactiflora* Pall. [Paeoniaceae]), and Phellodendri chinensis cortex (*Phellodendron chinense* C.K.Schneid. [Rutaceae]). This Traditional Chinese formula has been widely used to prevent early signs of ageing in Chinese society for centuries, particularly helpful for the treatment of neurodegenerative diseases in older, such as Alzheimer's disease (Kang et al. 2021), dementia (Qin et al. 2019), vertigo, and presbycusis (Wei et al. 2017). The clinical trial has shown that the total effective rate of clinical treatment of deafness with YQCM decoction is 89.19%, and a follow-up survey of patients with significant effects has been carried out and no recurrence was found (Wei et al. 2017). Similar final efficiency rates were reported as well in the clinical trial conducted by Li (Li 2013). *Ginseng radix et rhizoma* and *Astragali radix*, the main components of YQCM, have been demonstrated to improve hearing thresholds in patients with sensorineural hearing loss and alleviate the symptoms of tinnitus (Xiong et al. 2012; Doosti et al. 2014). Further *in vitro* and animal studies revealed that *Ginseng radix et rhizoma* and *Astragali radix* can reduce oxidative damage in the cochlea by inhibiting reactive oxygen species (ROS) and nitric oxide synthase (NOS) production in the organ of Corti and SGNs and auditory hair cells (Choung et al. 2011; Xiong et al. 2011; Tian et al. 2014). At present, most of the studies on YQCM decoction in the prevention and treatment of ARHL are from clinical trials, but the molecular mechanisms underlying the therapeutic effects of this prescription remain unclear. Studies based on molecular targets and related signal pathways of this prescription action on ARHL are limited. Thus, further research with the appropriate approaches is needed to comprehensively reveal the involved potential mechanisms, which will provide a scientific basis for clinical trial research and product development of YQCM decoction. In this paper, we construct a multidimensional network of component-target-pathway-disease by using network pharmacology. Gene Ontology (GO), and pathway enrichment analyses were performed to further reveal the biological mechanism of action of YQCM against ARHL. In addition, molecular docking and *in vitro* experiments were used to confirm the key mechanism for its action. The findings may provide new reference information for the further development and application of YQCM. The idea of this study is shown in the flow chart in Figure 1.

## Materials and methods

### Identify the chemical constituents of YQCM decoction

The chemical composition information of eight traditional Chinese medicines in YQCM decoction was obtained by using the Traditional Chinese medicine systems pharmacology database and analysis platform, TCMSP Version 2.3. Two parameters, oral bioavailability (OB) and drug-likeness (DL) were used to screen the above compounds, and the chemical components with both OB ≥ 30% and DL ≥ 0.18 were selected as potentially effective chemical components.

### Prediction of targets and construction of the visual network of ingredient-action target

Based on the identified components, the corresponding targets were predicted by DrugBank online database (access: Feb 17, 2020, https://go.drugbank.com;). We used “Age-related hearing loss” and “Presbycusis” as the keywords in the GeneCards database (access: Feb 25, 2020, https://www.genecards.org) and Online Mendelian Inheritance in Man database (access: Feb 25, 2020: OMIM database, https://www.omim.org) to search potential targets of ARHL. The visualisation network of the ingredient-action target was established by Cytoscape 3.6.0. Then the Network Analyser plug-in is used to analyse the network diagram to find its main active components and important targets.

### Construction of protein-protein interaction (PPI) network and enrichment analysis

The common targets of disease and drug are obtained by using the Venny online tools, that is, the potential target of YQCM decoction in the prevention and treatment of presbycusis. The drug-disease common target was introduced into the STRING: functional protein association networks. The PPIs with medium confidence were included in this study. Subsequently, a PPI network for the potential target was constructed using Cytoscape. The "Network Analyser" plug-in of Cytoscape software is used to analyse the topological attributes of the PPI network. The topological parameters, including Degree, Betweenness, and Closeness, were applied for screening core targets. To explore the mechanism underlying YQCM’s effect on ARHL, the Kyoto Encyclopaedia of Genes and Genomes (KEGG) pathway and GO including cellular components (CC), biological processes (BP), and molecular functions (MF) were analysed by the database for annotation, visualisation, and integrated discovery (DAVID) tools (version 6.8, https://david.ncifcrf.gov). The statistical significance threshold of enrichment analysis was established at *p* < 0.05.

### Molecular docking

The crystal structures of the target were retrieved from the RCSB (https://www.rcsb.org/) database. Three-dimensional structures of the target were retrieved from the PubChem database and minimised using MMFF94 forcefield. The water molecules and original ligands were removed by PyMol 2.5 software and transferred to pdbqt format via ADFR suite 1.0. Subsequently, AutoDock Vina 1.1.2 was employed for molecular docking to evaluate the binding affinity between core potential targets and core compounds. The binding activity is evaluated by the binding energy (kJ/mol). The smaller the binding energy, the more stable the docking module. The minimum binding energy <−5.0 kcal/mol indicates a good binding affinity between receptor and ligand (Yang et al. 2021; Zhang et al. 2021).

### Molecular dynamics simulation

After docking, the molecular dynamics (MD) simulation strategy was carried out to simulate the binding stability of the receptor and ligand by the AMBER 18 software package (Park et al. 2016). The atomic partial charges of the ligands were obtained by the restrained electrostatic potential fitting method (Wang et al. 2000) based on the electrostatic potentials computed at the Hartree-Fock SCF/6-31G* level of theory. Subsequently, the AMBER force field ff14SB (Maier et al. 2015) and GAFF2 field were applied for describing protein and chemical ligand. The complex system was immersed into a rectangular periodic box of pre-equilibrated TIP3P water at least 10 Å distance around the complexes. Finally, an appropriate amount of sodium ions was added to maintain the electroneutrality of the simulation system.

For each simulation, a sophisticated protocol is followed, including minimisation, heating, equilibration, and production. Initially, the water molecules were minimised through 2500 steps of steepest descent followed by 2500 steps of conjugate gradient while proteins were kept at the position except for the hydrogens. The same minimisation protocol was applied to optimise the side chains. Then, the whole system was relaxed for 5000 steps without any restraints. After energy minimisation, each system was gradually heated at a constant volume from 0 K to 300 K over a coupling time of 100 ps with position restraints. After heating, the whole system was equilibrated over 100 ps at a constant pressure of 1 bar. Subsequently, another 100 ps pre-equilibration was performed for pressure relaxation with a weak restraint on the protein backbone. After that, a 100 ns MD simulation was conducted for each system to produce trajectories. During MD simulations, periodic boundary conditions were employed and the direct space interaction was calculated by using the Particle-Mesh Ewald (PME) method with long-range electrostatic interaction. The SHAKE algorithm was used to restrict the bond to the hydrogen atom (Kräutler et al. 2001). The time-step was set at 2 fs and the trajectory was saved every 10 ps for subsequent analysis. The MMPBSA.py module in AMBER 18 was used for Molecular Mechanics/Generalized Born Surface Area (MM/GBSA) calculations based on the MD trajectory (Miller et al. 2012).

### Preparation of YQCM decoction

Astragali Radix, Ginseng Radix et Rhizoma, Glycyrrhizae Radix et Rhizoma, Cimicifugae Rhizoma, Puerariae Lobatae Radix, Viticis Fructus, Phellodendri Chinensis Cortex, Paeoniae Radix Alba, purchased from Kangqiao Chinese Medicine Tablet Co., Ltd, were used to prepare YQCM decoction with the proportions as 10:10:10:6:6:3:2:2. All crude drugs were soaked in 60% ethanol for 0.5 h and then extracted with 60% ethanol 2 times for 1.5 h, respectively. Next, the ethanol extract was evaporated *in vacuo* until the relative density was 1.15. The YQCM powder was obtained by a freeze-dryer and stored at −20 °C for later use. Liquid Chromatography-Mass Spectrometry was conducted on an LTQ Orbitrap XL™ hybrid FT mass spectrometer (ThermoFisher, Waltham, MA, USA). Liquid chromatographic separation was performed on an ACQUITY UPLC^®^HSS T3 1.8 μm 2.1 × 100 mm column maintained at 35 °C and eluted with gradient water. The mixture containing six reference components was used for the qualitative analysis: (1) puerarin, (2) ginsenoside-Rg1, (3) luteolin, (4) astragaloside IV, (5) kaempferol, and (6) formononetin.

### Cell culture

HEI-OC1 cells (the House Ear Institute-Organ of Corti 1 cell line) in high-glucose Dulbecco's modified Eagle's medium (DMEM) containing 10% foetal bovine serum without antibiotics. Cell cultures were maintained at 33 °C in a humidified incubator with 10% CO_2_.

### Cell viability

Cells were incubated in a 96-well plate at a density of 5 × 10^3^ cells per well for 24 h. To investigate the cytotoxic effect on YQCM, cells were incubated with YQCM solutions with different concentrations (0, 50, 75, 100, 125, 250, 500, and 1000 μg/mL) for 24 h. To evaluate YQCM’s protective effect under oxidative stress, cells were replaced with fresh DMEM containing different concentrations of YQCM (0, 1, 10, 30, 60, 90, and 120 μg/mL) for 24 h. The positive control group and the YQCM-treated groups were then exposed to H_2_O_2_ (100 μM) for 3 h. After various treatments, 10 μL of the cell counting kit-8 (Beyotime, Shanghai, China) solution was added to each well of the plate. Cell viability was quantified at an absorbance of 450 nm using a VICTOR^®^ Multimode Microplate Reader (Perkin Elmer, Waltham, MA, USA).

### High-content analysis of mitochondrial membrane potential

HEI-OC1 cells were plated in a 96-well plate and exposed to different concentrations of YQCM (0, 10, 30, and 90 μg/mL) for 24 h as described in “Cell viability.” The positive control group and the YQCM-treated groups were then exposed to H_2_O_2_ (100 μM) for 3 h. To monitor the mitochondrial membrane potential, the fluorescent TMRE (ThermoFisher, Waltham, MA, USA) was added to each well for 15 min and then incubated in 1 μg/mL Hoechst 33342 (Beyotime, Shanghai, China) for 10 min. Subsequently, the plate was washed with PBS/0.2% BSA. The TMRE fluorescent signal was immediately scanned and quantified with the High-content imaging system ImageXpress Micro microscope (Molecular Devices, Sunnyvale, CA, USA) using a 20 × objective to take an image covering the whole well for each well.

### Western blot

Proteins were extracted from treated cells and put through the 12% Protein Gel electrophoresis. After that, protein samples were transferred into the polyvinylidene fluoride membrane (Merck Millipore, Darmstadt, Germany) and blocked for 15 min with QuickBlock solution (Beyotime, Shanghai, China). Afterward, the blotted membrane was incubated with specific primary antibodies: anti-γH2AX (#9718, 1:1000, Cell Signalling Technology, USA), anti-cleaved caspase-3 (ASP175) (#9661,1:1000, Cell Signalling Technology, Danvers, MA USA), anti-β-actin (#4970, 1:1000, Cell Signalling Technology, Danvers, MA) for overnight at 4 °C. Then, each membrane was washed with TBS-T buffer and incubated with a secondary antibody for 1 h at room temperature. The blots were visualised by ECL Plus Western Blotting Substrate (Perkin Elmer, Waltham, MA, USA) and imaged by ChemiDOC^TM^ Touch Imaging System (Bio-Rad, California, Hercules, USA) using standard chemiluminescence. Quantification of band intensity was analysed with Image Lab Software (Bio-Rad, California, Hercules, USA).

### Statistics analysis

All statistical analyses were performed by GraphPad 7.0 (GraphPad Software, San Diego, USA). One-way analysis of variance (ANOVA) followed by Dunnett’s *post hoc* test was applied for multiple group comparisons. *P* values < 0.05 were considered statistically significant. Data are expressed as the means ± standard deviation (SD).

## Results

### Chemical compounds in YQCM decoction

Using the TCMSP database, 87 compounds were found in Astragali radix, 190 in Ginseng radix et rhizoma, 280 in glycyrrhizae radix et rhizome, and 187 in cimicifugae rhizome, 18 in Puerariae lobatae radix, 144 in Viticis fructus, 140 in Phellodendri* chinensis cortex*, 85 in Paeoniae radix alba. Among them, 206 compounds met the criteria of OB ≥ 30% and DL ≥ 0.18.

### Prediction of targets and construction of a visual network of YQCM ingredient-action target

**Figure 2.**
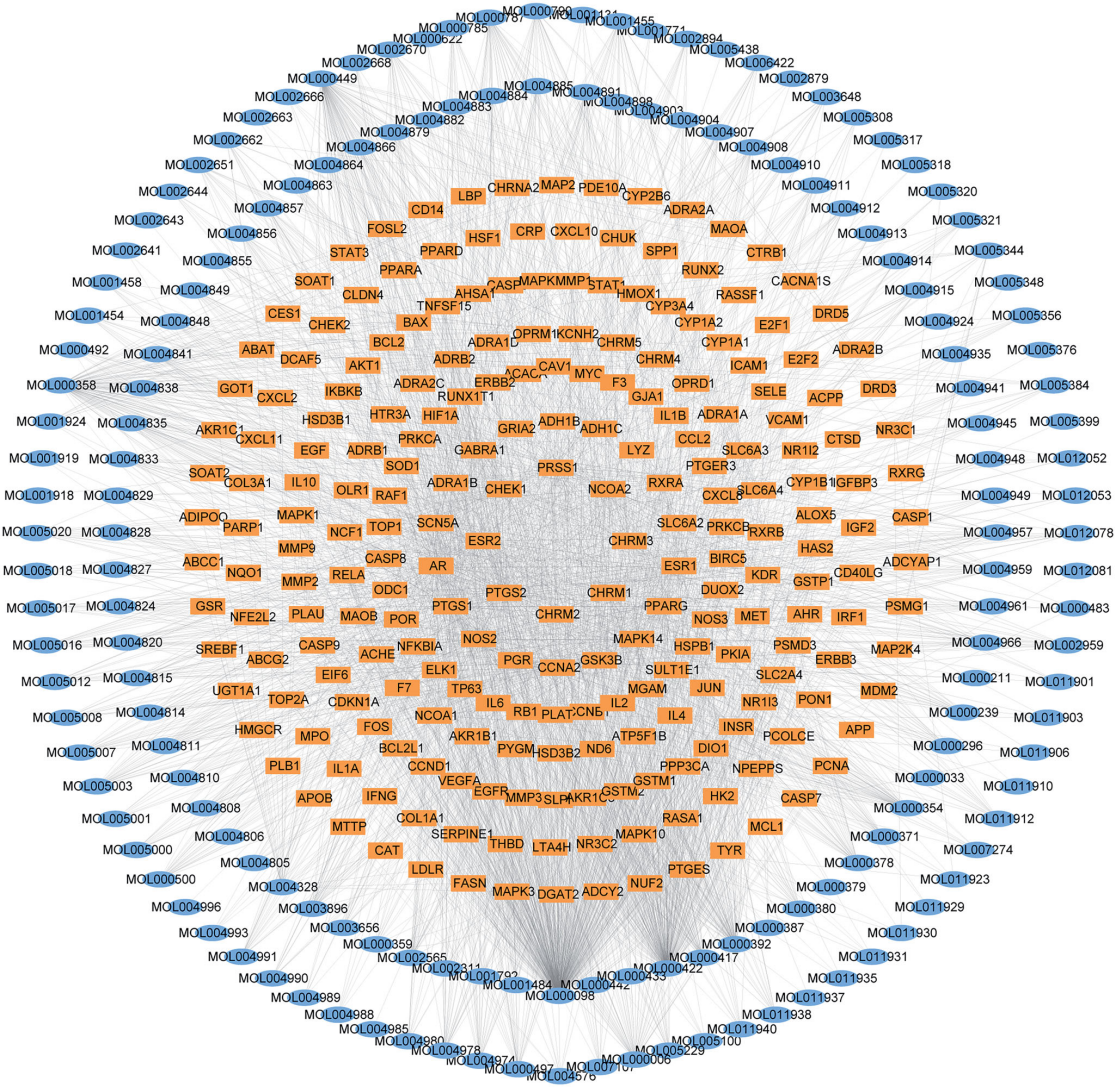
Network of the ingredient-action target. The blue oval cycle is on behalf of the active compound from YQCM. The yellow squares represent the compound’s target.

206 potential bioactive compounds were further searched on the Drugbank and TCMSP platform for target prediction and 233 corresponding potential targets were obtained. To illustrate the relationship between the compounds and their targets, a compound-target network was established via Cytoscape 3.6.0 (Figure 2). 395 nodes (162 nodes for candidate bioactive ingredients and 233 nodes for potential targets), as well as 2813 edges, were found in the network. According to topological analysis, the top 10 with the highest degree of nodes were quercetin, kaempferol, β-sitosterol, stigmasterol, formononetin, luteolin, isorhamnetin, fumarine, 7-*O*-methylisomucronulatol, 7-methoxy-2-methyl isoflavone (Table 1), which might be involved in the regulation of multiple ARHL targets.

**Table 1.** Characters of core compounds in YQCM decoction.

Mol ID	Mol name	BC	CC	Degree
MOL000098	Quercetin	0.406	0.511	544
MOL000422	Kaempferol	0.083	0.423	255
MOL000358	beta-Sitosterol	0.027	0.398	104
MOL000449	Stigmasterol	0.036	0.400	104
MOL000392	Formononetin	0.026	0.388	84
MOL000006	Luteolin	0.088	0.413	50
MOL000354	Isorhamnetin	0.019	0.394	48
MOL000787	Fumarine	0.008	0.378	38
MOL000378	7-*O*-Methylisomucronulatol	0.012	0.402	32
MOL003896	7-Methoxy-2-methyl isoflavone	0.012	0.402	31

Betweenness Centrality: BC; Closeness Centrality: CC

### ARHL-related gene

**Figure 3.**
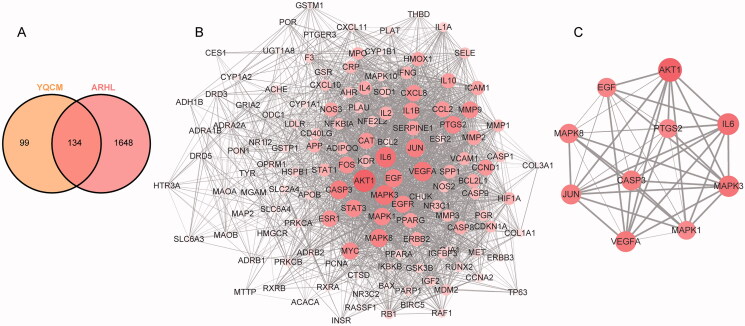
YQCM’s therapeutic targets for ARHL and their Protein-Protein interactions. (A) 134 common targets shared with YQCM and ARHL were obtained by the Venn map. (B) The PPI network of potential targets of ELZC working on ARHL was acquired from the STRING database. (C)The interaction between the top 10 hub targets was visualised by Cytoscape software. In the network, the colour and size of nodes reflected degree centrality, while the edge thickness reflected the combined score.

The keyword “Age-related hearing loss” and “Presbycusis” were used to search the reported ARHL-related genes in the two disease databases OMIM database and GeneCards database. A total of 1782 genes were obtained after removing duplicates. Common target genes between YQCM decoction and ARHL were acquired by overlapping the above targets via the Venn diagram. A total of 134 common targets were obtained as shown in Figure 3A.

### Protein-Protein interaction network

Common target genes between YQCM decoction and ARHL were input into the STRING database. The protein-protein interaction of these common targets was constructed as a PPI network, in which the greater degree of the node, the greater the role of the target in the network. According to the network topology properties, 52 targets with a degree greater than the median (Median of Betweenness = 0.0026; Median of Closeness = 0.5624; Median of Degree = 32) were predicted as the core targets of YQCM action on ARHL. In this network, AKT1, IL-6, MAPK3, VEGFA, CASP3, JUN, MAPK8, EGF, PTSG2, and MAPK1 were the top 10 nodes in terms of degree value (Table 2).

**Table 2.** Characters of ten core therapeutic targets.

No.	Target name	BC	CC	Degree	UniProt
CT-01	AKT1	0.072	0.831	106	P31749
CT-02	IL6	0.037	0.778	95	P05231
CT-03	MAPK3	0.038	0.764	92	P27361
CT-04	VEGFA	0.022	0.756	90	P15692
CT-05	CASP3	0.025	0.735	85	P42574
CT-06	JUN	0.018	0.735	85	P05412
CT-07	MAPK8	0.018	0.723	82	P45983
CT-08	EGF	0.018	0.719	81	P01133
CT-09	PTGS2	0.014	0.711	79	P35354
CT-10	MAPK1	0.015	0.707	78	P28482

Betweenness Centrality: BC; Closeness Centrality: CC

### Enrichment analysis of gene ontology and KEGG pathway

**Figure 4.**
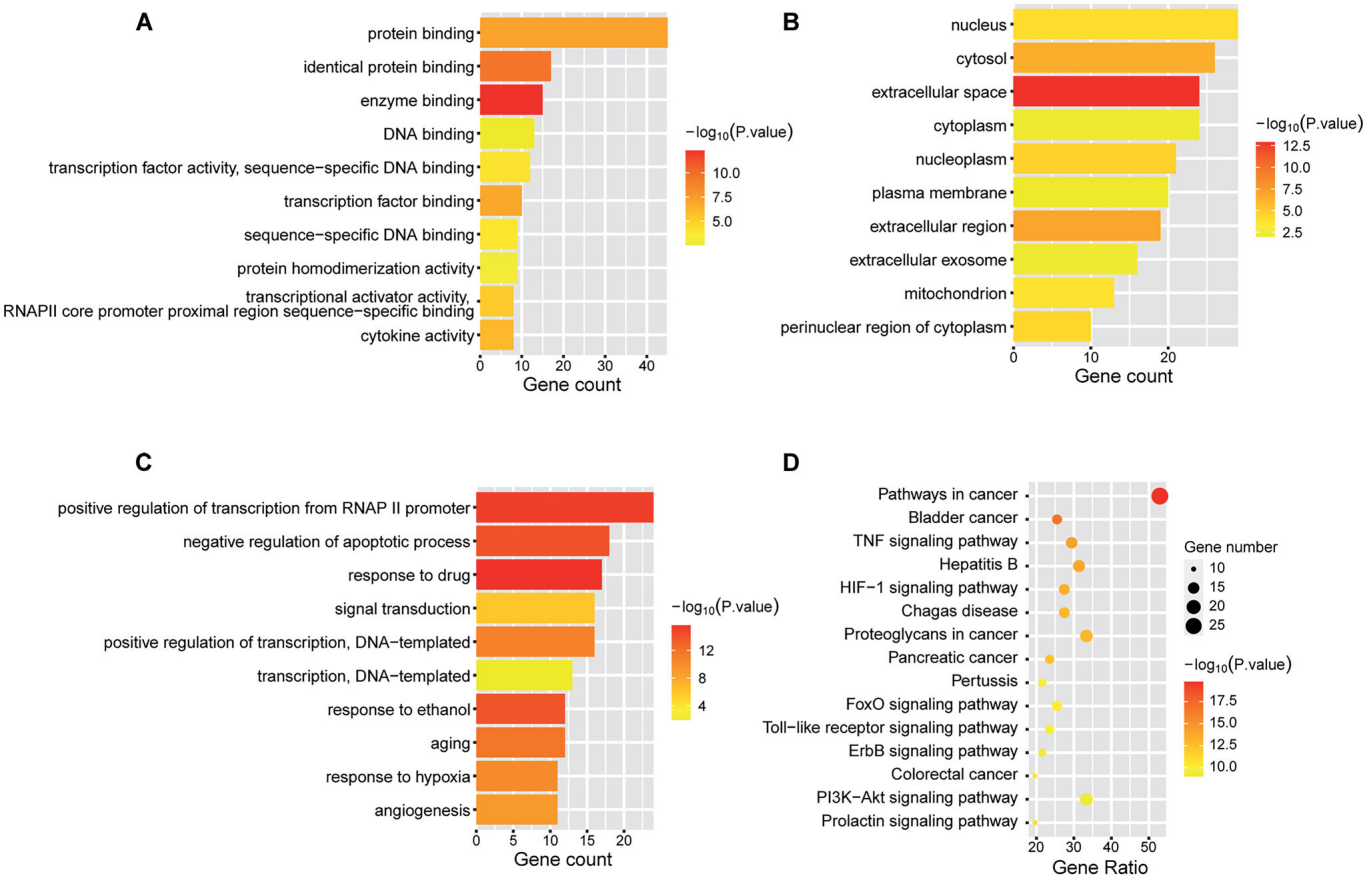
Enrichment analysis of Gene ontology and KEGG pathway. 52 targets with a degree greater than the median were applied for enrichment analysis. Gene Ontology including biological processes (A), cellular components (B), molecular functions (C), and KEGG pathway (D) was analysed by DAVID. The statistical significance threshold of enrichment analysis was established at *p* < 0.05.

To understand the function and the underlying significance of the YQCM’s therapeutic effect, the above 51 core targets were entered into the DAVID platform for GO term and KEGG pathways enrichment analysis. The results showed that the targets were mainly enriched in 45 molecular functions, 301 biological processes, and 28 cellular components. The top 10 enriched terms are shown in Figures 4A–C. We found that biological processes mainly concentrated on positive regulation of transcription from RNA polymerase II promoter, negative regulation of the apoptotic process, response to the drug, signal transduction, positive regulation of transcription, DNA-templated, response to ethanol, ageing, response to hypoxia, angiogenesis. The molecular functions were related to protein binding, enzyme binding, DNA binding, transcription factor binding, protein homodimerization activity, transcriptional activator activity, cytokine activity, etc. Finally, the cellular component was mainly composed of the nucleus, cytosol, nucleoplasm, extracellular space, cytoplasm, nuclear plasm, plasma membrane, extracellular region, mitochondrial, protein complex, and perinuclear region of cytoplasm, and so on. KEGG pathway analysis showed that the potential targets of YQCM action on ARHL were involved in 94 signalling pathways (*p* < 0.05). These pathways are predominantly involved in the HIF-1 pathway (consist of targets of CDKN1A, NOS3, EGF, STAT3, SERPINE1, HIF1A, EGFR, VEGFA, IL6, ERBB2, AKT1, MAPK1, HMOX1, MAPK3), FoxO (consist of targets of IL10, CDKN1A, EGF, STAT3, EGFR, IL6, MAPK8, CCND1, CAT, MDM2, AKT1, MAPK1, MAPK3) and PI3K/AKT pathway (consist of targets of GSK3B, CDKN1A, NOS3, EGF, EGFR, IL2, VEGFA, IL4, IL6, CCND1, MYC, MDM2, SPP1, AKT1, MAPK1, BCL2L1, MAPK3). The top 10 KEGG enrichment pathway ranking *p* values according to the order from small to large are displayed in Figure 4D.

### Molecular docking of main chemical compounds binding to hub genes

**Figure 5.**
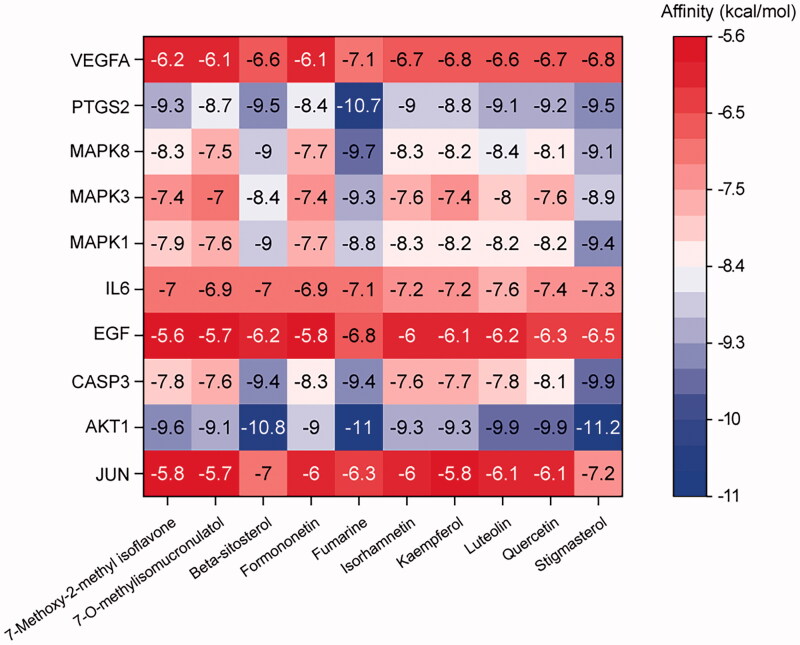
Heatmap of molecular docking results. The x-axis represents ten core active bioactive compounds; the y-axis represents the hub targets. The docking score represents the lowest binding energy of compounds-target molecular docking (kJ/mol).

The ten candidate therapeutic targets with high degree value, AKT1, IL6, MAPK3, VEGFA, CASP3, JUN, MAPK8, PTSG2, EGF, and MAPK1, were docked with the ten core active compounds of YQCM. The lowest binding energy of the molecular docking of the core targets and compounds was presented in the heatmap as shown in Figure 5. We found all docking values were lower than −5 kcal/mol, indicating a stable combination between core targets and core compounds (Yang et al. 2021; Zhang et al. 2021). In particular, AKT, PTSG2, and CASP3 were found to have a strong binding affinity with all ten key compounds, values ranging from −7.6 to −11.2 kcal/mol.

### Molecular dynamics simulation

**Figure 6.**
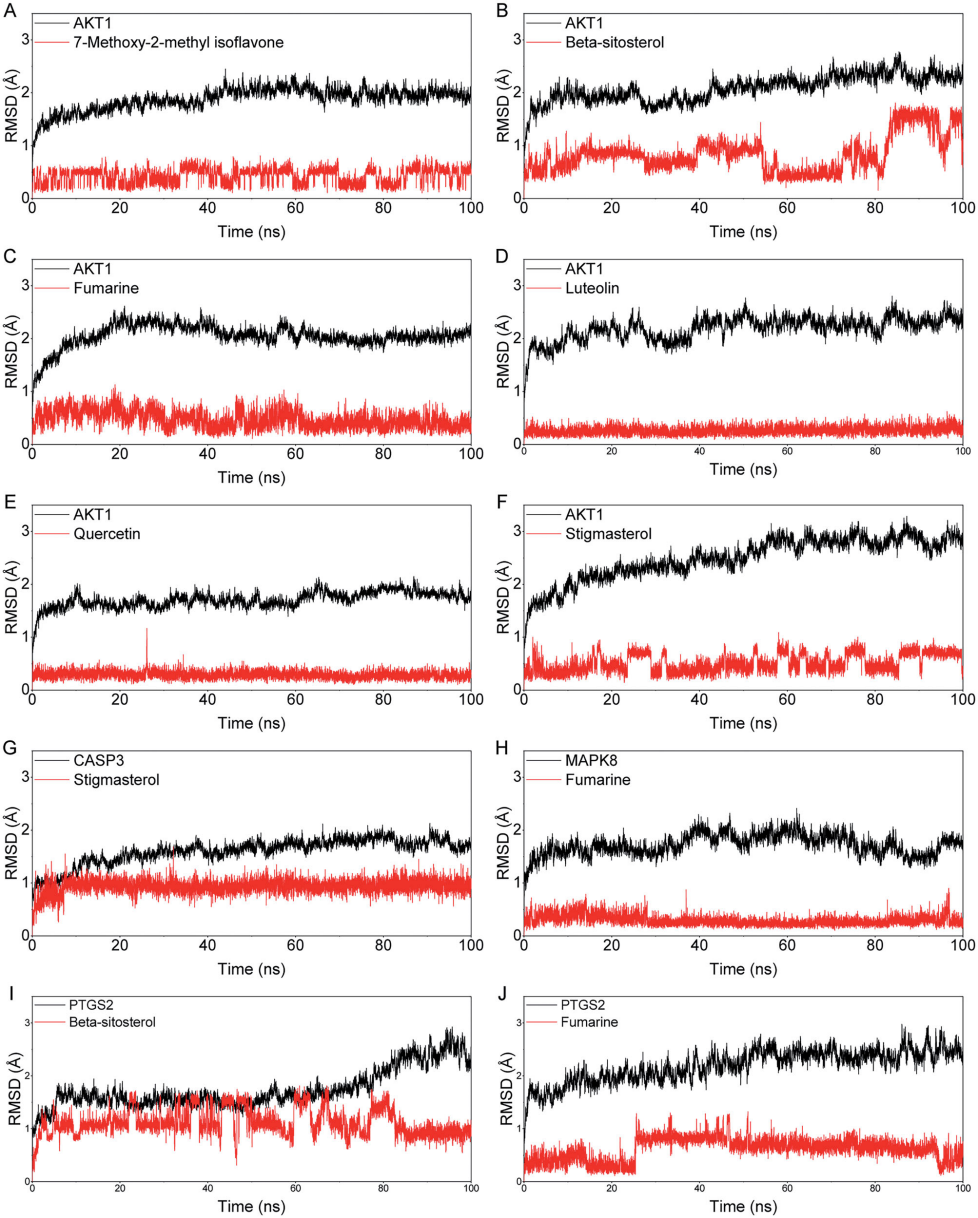
The picture of the RMSD value of the molecular complex system in 100 ns time interval. The dynamic stability of AKT1 with 7-Metho-2-met_isoflavone (A), AKT1 with β-sitosterol (B), AKT1 with fumarine (C), AKT1 with luteolin (D), AKT1 with quercetin (E), AKT1 with stigmasterol (F), CASP3 with stigmasterol (G), MAPK8 with fumarine (H), PTGS2 with β-sitosterol (I), PTGS2 with fumarine (J).

Molecular dynamics simulation was used for studying the binding stability of the top ten protein-compound with the best molecular docking scores. The MM/GBSA method was further used to calculate the binding energies. The specific binding free energy of AKT1_7-Metho-2-met_isoflavone, AKT1_β-sitosterol, AKT1_fumarine, AKT1_luteolin, AKT1_quercetin, AKT1_stigmasterol, CASP3_stigmasterol, MAPK8_fumarine, PTGS2_β-sitosterol, PTGS2_fumarine was showed in Table 3. The highest binding free energy combinations were CASP3_Stigmasterol (−42.509 kcal/mol) and AKT1_β-sitosterol (−39.354 kcal/mol), followed by AKT1_Stigmasterol (−39.024 kcal/mol). And the MD simulation indicated that the combination of AKT and the main chemical compounds was relatively stable. Our analysis showed that these high binding free energy combinations were mainly contributed by van der Waals energy, electrostatic energy, and non-polar energy. The root-mean-square deviation (RMSD) curve represents the stability of the protein conformation. The RMSD pattern of protein and molecule during the 100 ns simulations was shown in Figure 6. As can be seen, the RMSD values of the black curve (protein) were maintained below 3.0 Å and the RMSD values of the red curve (molecule) were less than 2.0 Å, indicating the stability of the docked complex (Ramirez and Caballero 2018). It is worthy to note that the complexes of AKT1_luteolin (Figure 6D), AKT1_quercetin (Figure 6E), CASP3_stigmasterol (Figure 6F), and MAPK8_fumarin (Figure 6H) possessed a nonfluctuating RMSD curve during MD, which indicates that the binding between the protein and the molecule is extremely stable.

**Table 3.** Binding free energies and energy components are predicted by MM/GBSA (kcal/mol).

System name	ΔE_vdW_	ΔE_elec_	ΔG_GB_	ΔG_SA_	ΔG_bind_
AKT1/7-Metho-2-met_isoflavone	−35.687	−1.434	16.212	−4.588	−25.497
AKT1/Beta-sitosterol	−63.367	−4.602	36.229	−7.615	−39.354
AKT1/Fumarine	−47.073	−10.635	37.235	−5.103	−25.581
AKT1/Luteolin	−36.229	−12.946	32.272	−4.649	−21.552
AKT1/Quercetin	−33.796	−82.818	96.019	−4.891	−25.486
AKT1/Stigmasterol	−51.843	−5.429	24.333	−6.085	−39.024
CASP3/Stigmasterol	−50.238	−2.783	16.606	−6.095	−42.509
MAPK8/Fumarine	−49.891	−48.479	70.778	−5.562	−33.157
PTGS2/Beta-sitosterol	−49.129	−7.473	27.628	−5.840	−34.814
PTGS2/Fumarine	−41.133	−66.329	85.504	−4.831	−26.788

ΔE_vdW_: van der Waals energy.

ΔE_elec_: electrostatic energy.

ΔG_GB_: electrostatic contribution to solvation.

ΔG_SA_: non-polar contribution to solvation.

ΔG_bind_: binding free energy.

### Experimental validation

**Figure 7.**
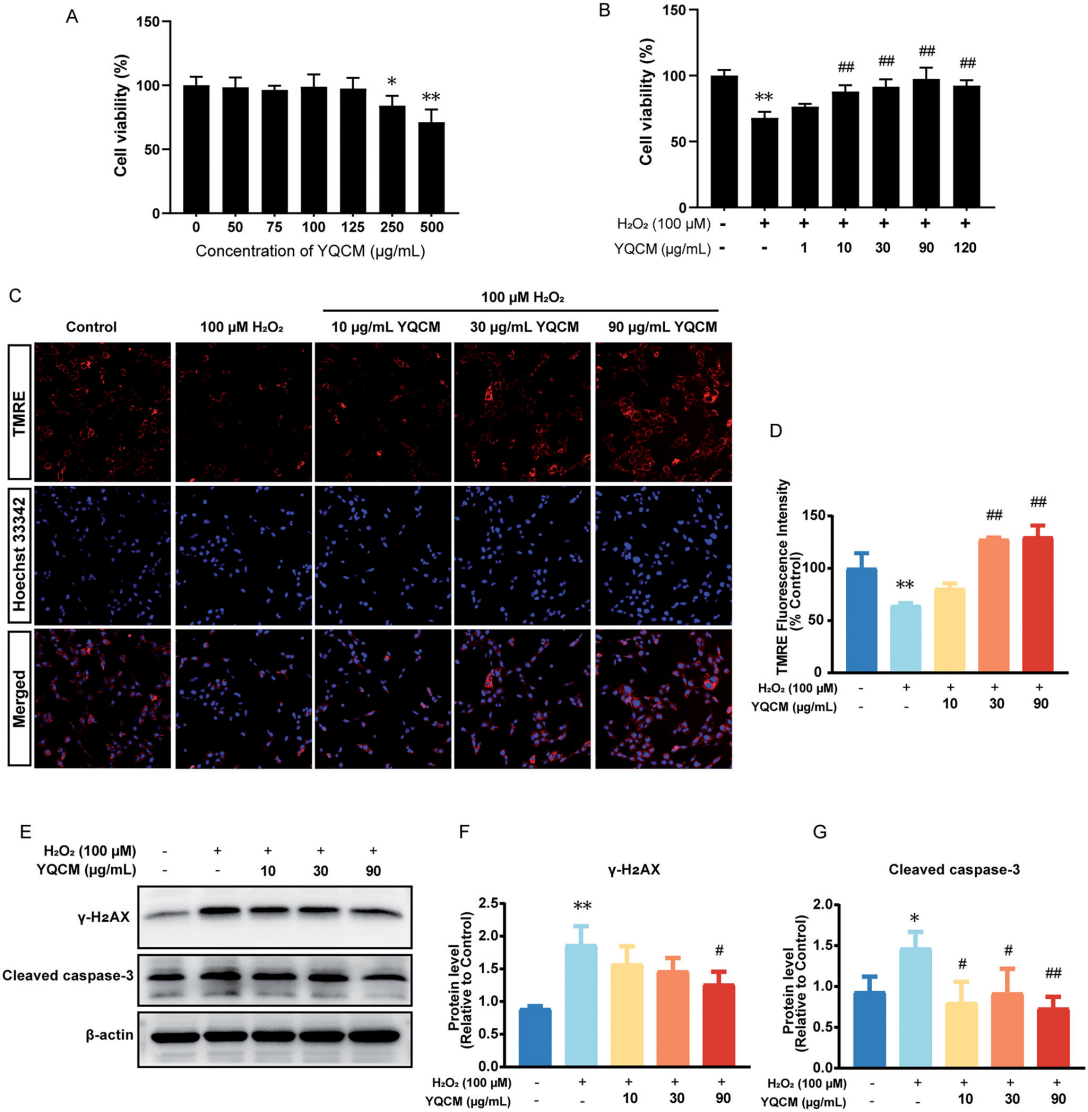
YQCM decoction exhibits anti-apoptotic activity, protecting Hei-oc1 cells from DNA damage induced by H_2_O_2_. (A) HEI-OC1 cells were incubated with YQCM (0, 50, 75, 100, 125, 250, and 500 μg/mL) for 24 h, and then cell viability was determined by CCK8 assay. (B) Cells were exposed to 100 μM H_2_O_2_, and cell viability with and without YQCM pre-treatment under oxidative conditions was determined by CCK-8 assay as well. (C and D) Cells were pre-treated with YQCM (10, 30, 90 μg/mL) for 24 h, and the mitochondrial membrane potential of cells with and without YQCM retreatment under oxidative conditions were examined by TMRE immunofluorescence. The fluorescence intensity was quantified with the High-content imaging system (*n* = 3). (E–G) Western blotting of DNA-damage marker, γH2AX, and apoptosis protein, cleaved caspase-3 (*n* = 3). The values represent the means ± SD, *p < 0.05 and **p < 0.01 vs. control, #*p* < 0.05 and ##*p* < 0.01 vs. H_2_O_2_ (Analysis of variance (ANOVA), using Dunnett’s *post hoc* test).

To evaluate YQCM’s cytotoxicity on the HEI-OC1 cells, cells were treated with 0, 50, 75, 100, 125, 250, and 500 μg/mL of YQCM for 24 h. As displayed in Figure 7A, YQCM did not show cytotoxicity up to 125 μg/mL. Next, we investigated the cell viability with and without YQCM pre-treatment under oxidative conditions. The results revealed that compared with the control group, the viability of cells exposed to H_2_O_2_ (100 μM) was significantly reduced by 68% (Figure 7B). However, YQCM ( 10, 30, and 90 μg/mL) showed a concentration-dependent recovery of cell viability, which were 88.07, 91.73, and 97.45%, respectively. Mitochondrial membrane potential is a widely accepted indicator for the apoptotic process. It will decrease during apoptosis because of the opening of the mitochondrial permeability pores and the loss of the electrochemical gradient. As shown in Figures 7C and D, the ΔΨM of the H_2_O_2_-treated group dropped by nearly 40% as compared with the control group (*p* < 0.01). On the contrary, YQCM pre-treatment attenuated the H_2_O_2_-induced reduction of ΔΨM in a concentration-dependent manner. The essential proteins responsible for DNA damage (γ-H2AX) and apoptosis (cleaved caspase-3) were measured in H_2_O_2_-induced HEI-OC1 pre-treated with or without YQCM. As shown in Figures 7E and F, the protein level of γ-H2AX in HEI-OC1 cells with H_2_O_2_ increased by 2-fold and decreased by 33% after 90 μg/mL YQCM treatment. Immunoblotting results showed that exposure to H_2_O_2_ for 3 h resulted in a 50% increase in the expression of cleaved caspase-3. There is a 37% decrease in cleaved caspase-3 level at 30 μg/mL and a 50% decrease at 90 μg/mL YQCM (Figure 7E–G).

## Discussion

Age-related hearing loss always exhibits a mixture of several pathological changes within the sensory hair cells, auditory neurons SGNs and stria vascularis (Keithley 2020). With the characteristics of multiple targets, Chinese herbal medicines may be beneficial to the treatment of ARHL. Preliminary clinical observations have shown that YQCM and its herbs may restore hearing loss in the elderly (Li 2013; Wei et al. 2017). These data raised the question of what underlying molecular mechanisms could explain YQCM’s protective effect on hearing function? To elucidate it, we attempted to use network pharmacology to show the interaction of the bioactive compounds, target molecules, and biological functions.

In our network pharmacology study, 206 potential compounds retrieved from YQCM were considered pharmacokinetically active and probably absorbed and distributed in the human body. The top 10 compounds with high degrees were quercetin, kaempferol, β-sitosterol, stigmasterol, luteolin, isorhamnetin, fumarine, 7-*O*-methylisomucronulatol, 7-methoxy-2-methyl isoflavone. Quercetin and kaempferol, the dominant flavonoid found generally in plants, have been reported to exhibit antioxidant and anti-inflammatory effects (Di Petrillo et al. 2022). Quercetin-mediated oxidative stress clearance and anti-apoptosis have been proven to attenuate noise-induced hearing loss and protect against hair cell loss induced by neomycin (Hirose et al. 2016). Many research groups have reported flavonoid luteolin’s protective effect on heart function via blocking oxidative stress, decreasing inflammation, ameliorating apoptosis, and up-regulating autophagy (Luo et al. 2017). Similar protective effects on auditory function were observed recently. Choi et al. (2008) firstly tested the luteolin for its antioxidant activity using the auditory hair cell HEI-OC1 *in vitro*. This study indicated that luteolin markedly enhances HO-1 expression via activation of ERK, thereby blocking cisplatin-induced oxidative damage and apoptosis. Another study showed luteolin effectively protects against oxidative stress-induced cellular senescence through p53 and SIRT1 in HEI-OC1 (Zhu et al. 2021). These results further support that luteolin’s protective effect on HEI-OC1 cells may help improve hearing function. β-Sitosterol and stigmasterol are two compounds that have received attention in recent years in ageing and neurodegenerative diseases for their ability to cross the blood-brain barrier and exhibit important activities related to removing Aβ plaque and reducing neuroinflammation (Sharma et al. 2021). The diverse and promising effects from these compounds might contribute to YQCM decoction as a potential therapeutic agent for age-related hearing loss.

In this study, AKT1, IL6, VEGFA, Caspase-3, JUN, MAPK8, PTGS2, EGF, and MAPK1 are the top 10 nodes with the highest degree value. It was speculated that they might be the key targets involved in YQCM against ARHL. We conduct molecular docking for main chemical compounds binding to these hub targets. The results showed that most of the core chemical compounds have good docking affinities with hub targets, especially AKT1, PTGS2, and Caspase-3. Notably, the RMSD curves of the complexes of AKT1_luteolin, AKT1_quercetin, and CASP3_stigmasterol did not fluctuate in MD simulation, indicating that the binding between the protein and the molecule is very stable. Akt has been considered a pivotal anti-apoptotic factor in many different cell death paradigms (Zheng et al. 2020). Many studies have reported that compounds from herbal medicine could interact with Akt resulting in beneficial neuroprotective effects. For instance, flavanones could activate Akt/PKB signalling pathway and show prosurvival characteristics in the cortical neurons (Vauzour et al. 2007). Akt also plays a critical role in the cochlea for its phosphorylation of numerous downstream proteins, including caspase-3, and contributes to hair cell death (Haake et al. 2009; Sha et al. 2010). Besides hair cell death, recent data suggest inflammation is also the cause of age-related hearing loss (Verschuur et al. 2012). The hub gene, PTGS2, encoding the enzyme of cyclooxygenase-2 (COX-2), which is an important immune mediator, plays an imperative role in the ageing process. A recent study by Uraguchi et al. (2021) analysed 84 immune-related genes expression in the cochlea of 12-month-old mice with ARHL as compared to the young control mice. They found around 40% of immune-related genes were upregulated in the aged cochlea, including PTGS2. And PTGS2 were immunolocalized ubiquitously in aged cochlear structures, including the lateral wall (the stria vascularis and spiral ligament).

Notably, in this study, KEGG enrichment results showed that HIF-1, FoxO, and PI3K/AKT pathways were significantly associated with YQCM’s action on ARHL. It has been reported that HIF-1 pathways are closely related to the occurrence and development of age-related hearing loss. Cochlear hypoxia-inducible factor-1alpha (HIF-1α) levels were dramatically increased in the age-related hearing loss mice model (Riva et al. 2007; Hwang et al. 2013; Lin et al. 2017), especially in the spiral ganglion and the stria vascularis. Activation of HIF-1 might be related to cochlear injury induced by free radicals and appeared to be responsive to regulating target genes, involved in pathways of apoptosis with the caspase-3 protein (Hwang et al. 2013; Deal et al. 2017). In contrast, a recent study by Pak et al. (2019) revealed that CoCl2 pre-treatment protected auditory hair cells from H_2_O_2_-mediated cell death via the activation of the HIF-1α. This study proved that HIF-1 could ameliorate oxidative stress via redox-sensitive transcription factors nuclear factor erythroid 2-related factor 2 (Nrf-2), and improve an antioxidant enzyme peroxiredoxin 6 (Prdx6) in preventing auditory cell damage during ARHL. FoxO is a well-studied pathway affected by ototoxic drugs, which is one of the factors in increasing ROS production in ARHL. For example, the FoxO signalling pathway was activated following amikacin-induced ototoxicity which is consistent with increased apoptosis (Liu et al. 2017), or neomycin-induced ototoxicity which is related to decreased expression of antioxidant enzymes (Liu et al. 2016). Although the direct evidence that FoxO responds to age-related hearing loss is sparse, accumulating evidence indicates that misregulation of the FoxO signalling pathway may underlie both age-associated functional decline involving oxidative stress resistance, DNA damage repair, and apoptosis. Our KEGG analysis also predicted another pathway, PI3K/AKT, as the main pathway underlying the mechanism of YQCM’s protection effect on ARHL. During the past decade, emerging evidence has indicated that PI3K/AKT is the main upstream pathway for preventing and rescuing cochlea injury in maintaining cellular oxidative homeostasis via enhancing the downstream antioxidant factors (Tomobe et al. 2012; Zhu et al. 2018; Liu et al. 2021), downregulating the expression of proteins responsible for apoptosis (Kucharava et al. 2019; Zhang et al. 2021), etc.

Through enrichment analysis on the GO terms, we found that the therapeutic targets showed a strong correlation with the biological processes of negative regulation of the apoptotic process. 35% of core therapeutic targets (18/51) of YQCM were enriched in this process, including AKT1, GSK3B, CDKN1A, CASP3, MYC, CAT, BCL2L1, etc. Increasement of ROS production in ARHL, in addition to reduced antioxidant activity with age, will exacerbate the ROS-antioxidant imbalance (Tavanai and Mohammadkhani 2017). Furthermore, excessive ROS production adversely affects mitochondrial DNA (mtDNA), leading to extreme dysregulation in oxidative phosphorylation, further increasing ROS production (Chen and Tang 2014). Ultimately, the higher production of ROS may change DNA structure, resulting in cell death, including apoptosis, necrosis, and necroptosis (Tawfik et al. 2020) or cellular senescence (Menardo et al. 2012; Benkafadar et al. 2019), resulting in degenerative changes in the cochlear. To verify the results from GO function analysis, we set up a condition of oxidative stress in auditory hair cell culture with 100 μM H_2_O_2_. Pre-treatment of YQCM increased cell viability in HEI-OC1 cells exposed for 3 h to H_2_O_2_. Phosphorylation of the Ser-139 residue of the histone variant H2AX, forming γH2AX, is a sensitive marker for the appearance of DNA double-strand breaks (Mah et al. 2010). In our study, the level of γH2AX was markedly raised in HEI-OC1 cells treated with 100 μM H_2_O_2_ for 3 h, while pre-treatment of YQCM can significantly reduce the γH2AX level (Figure 7E), indicating that the presence of YQCM could inhibit oxidative stress-induced DNA damage. YQCM also reduced HEI-OC1 cell's apoptosis under oxidative stress by improving mitochondrial membrane potential, decreasing the level of cleaved caspase-3, suggesting that the negative regulation of apoptosis might be involved in YQCM protecting cell death from oxidative stress. Although the detailed molecular pathways involved in modulating auditory hair cell death remain to be identified, our network pharmacology and experiment data suggest that YQCM decoction may be against DNA damage and apoptosis induced by oxidative stress.

## Conclusions

For the first time, network pharmacology and molecular docking strategy to were utilised to explore the underlying mechanism of YQCM in treating ARHL. Our network pharmacology data suggest that modulations of DNA damage and apoptosis might be the key protective role for YQCM decoction. Potential hub targets for YQCM such as AKT1, CASP3, and PTGS2 may be exploited as potential strategies. We used molecular docking analysis and MD simulation to validate the interaction between core targets and compounds. Additionally, the protective effect of YQCM on ARHL and its mechanism were supported by the evidence that YQCM could alleviate oxidative stress-induced auditory hair cell death via modulating DNA damage/apoptosis. Future experiments using molecular methods and animal models as well are needed to support the current findings. Nevertheless, the results of this study provide an important basis for further research on the mechanism of YQCM in ARHL. This study proved that bioinformatics analysis combined with molecular docking and cell model is a promising method to explore other possible pharmacological interventions of ARHL.
